# Supplementary material for: Genetic Manipulation of Desulfovibrio ferrophilus and Evaluation of Fe(III) Oxide Reduction Mechanisms
Source: Microbiol Spectr. 2022 Nov 29;10(6):e03922-22. doi: 10.1128/spectrum.03922-22 (PMC9769857; doi:10.1128/spectrum.03922-22)
Supplement: Supplemental file 1 — Supplemental material. Download spectrum.03922-22-s0001.pdf, PDF file, 0.5 MB [file spectrum.03922-22-s0001.pdf]

SUPPLEMENTARY INFORMATION

Genetic Manipulation of *Desulfovibrio ferrophilus* and Evaluation of Fe(III) Oxide Reduction Mechanisms

Toshiyuki Ueki[a*], Trevor L. Woodard[a], and Derek R. Lovley[a,b#]

[a]Department of Microbiology, University of Massachusetts-Amherst, Amherst, MA, USA

[b]Institute for Applied Life Sciences, University of Massachusetts-Amherst

[*]Present address: Electrobiomaterials Institute, Key Laboratory for Anisotropy and Texture of Materials (Ministry of Education), Northeastern University, Shenyang 110819, China.

[#]Address correspondence to Derek R. Lovley, dlovley@umass.edu

```
DFE_2355 -------------MSLVINNNLMAINAARNMGSAFSMLASSVSRLSSGLRVTSAADDAAGLAIRELMRSEIAAMHQGARN
DFE_2171 -------------MSLVINHNLMAMNAARNLTESYGRLSTSTRRLSSGLRVGTAADDAAGLAIRELMRSDIKTLHQGVRN
DFE_0942 -------------MSLVINNNLMAMNAARNLGNAYDALASSTNKLSSGLRINSAADDAAGLAIRELMRADISTLNQGVRN
DFE_0943 -------------MSLVINNNIVAMTAARNLGTAYDALSSSTNKLSSGLRITTAADDAAGLAVRELMRSDIAALDQGVRN
DFE_1910 MDAGTEAFMEESDMSMVINNNLMAMNAARNLSNSFGALSVSVRRLSSGLRIGTAADDAAGMAIREIMRSDIAALNQGVRN
DFE_0352 -------------MSLVINNNLMAANASRNLSQSYSALGTSIRRLSSGLRVGIAADDAAGLAVRELMRSDIAALGQGVRN
DFE_2105 -------------MSLVINNNLMAANAARNLSQSFGALGTSVRRLSSGLRIGTASDDAAGLAIRELMRSDIAALNQGVRN
                      **:***:*::* .*:**:  ::. *. *  :******:  *:*****:*:**:**::* :: **.**

DFE_2355 VNDAISLIQTADGAAQVIDEKLIRMKELAEQAATGTYNSDQRLIIDSEYQAMASEITRIANATDFNGIHLLNGNLSGEN-
DFE_2171 ANDAISMVQTADGALQVIDEKLIRMKELAEQAATGTYNSDQRLIIDSEYQAMASEITRIALATDFNQVHLLDGTLSGVVP
DFE_0942 ANDAISMIQTADGALQVVDEKLIRMKELAEQAATGTYSSAQRLIIDSEYQAMASEITRIANATDFNGIMLLDGNLSGTE-
DFE_0943 ANDAISMIQTADGALQVIDEKLIRMKELAEQAATGTYSSTQRLIIDSEYQAMASEITRIANATDFNGIYLLNGNLSS---
DFE_1910 ANDAISMLQTADGALAVIDEKLIRMKELAEQAATGTYNSDQRLILDSEYQAMASEITRIANATDFNGLYLLNGNLSS---
DFE_0352 ANDAISMIQTADGALQVIDEKLIRMKELAEQASTGTYNSDQRLIIDSEYQAMASEITRIANATDFNGIYLLNGNLSG---
DFE_2105 ANDAISMIQTADGALQVIDEKLIRMKELAEQGATGTYNSDQRLIIDSEYQAMASEITRIANATDFNGIYLLNGNLSS---
         .*****::******  *:*************.:****.* ****:*************** ***** : **:*.**.

DFE_2355 SAHVGSALVPTGPLKVHFGSGNDCSEDYYYISINNATASALGVGNQSA-----AGAGFSISTQSAAQFALDAIKNAIVSK
DFE_2171 TDHDGSGMQPTGPMKIHFGTGNDSSEDYYFISIGGATASHFGLGLGSDGAASNFVGGQTISTQSLAQAALAALDQAIISK
DFE_0942 ASHDGSGMSATGPLKVHFGTGNDSTEDYYYVTIGDCTASALGVGNSAAV----GSAGYTISTQSAAQSALDAINNAIVSK
DFE_0943 DTHTGSGLSSTGKMKIHFGTANDSAEDYYYVQIGNSTASALGVGNSAAA----GSAGFTISTQSAAQNALDAITNAIVSK
DFE_1910 STHDGSGLTPSGKMKIHFGTANDSAEDYYYIQIGNATASALGVGNQAV-----AGAGFAISTQSAAQNALDGINDAIVSK
DFE_0352 STHTGSGLVSSGKLKVHFGTGNDSAEDYYYVQINTSTASAMGVGNQAAS----TSAGFTISTQSAAQNALDGIKNAIVSK
DFE_2105 DTHTGSGLVSTGKMKVHFGTANDSSEDYYYIRIDTSTASALGVGNQATS----TSAGFTISTQSAAQNALDGIKNAIVSK
           * **.:  :* :*:***:.**.:****:: *. .*** :*:*  :        .* :***** ** ** .: :**:**

DFE_2355 DKIRANLGALENRLY-AVTNLEIMAENLQASESRISDVDVALEMTQFVRAQIMSQAAVAMLAQANSLPRMAMQLIGG-
DFE_2171 DKIRANLGALQNRLENTITNLQIQSENLQAAESRISDVDVALEMTEFVRNQILTQSATAMLAQANSLPRLAMQLIGGG
DFE_0942 DNIRAHLGAMQNRLSNTISNLEIQAENLQAAESRISDVDVATEMTEYSKQQIITQSATSMLAQANSLPQLALQLLGG-
DFE_0943 DNIRANLGALQNRLSNTVSNLEIQAENLQAAESQISDVDVATEMTEYSRQQIISQSATSMLAQANSLPQLALSLLG--
DFE_1910 DKIRAGLGAMQNRLENTITNLQIQAENLSASESRISDVDVALEMTNFVKQQILSQAATAMLAQANSLPRMAMQLLGG-
DFE_0352 DKIRAALGGIQNRLENTISNLQIQAENLQAAESRISDVDVSLEMTEFVRNQIMSQSAVAMLAQANSLPRMAMSLLGG-
DFE_2105 DKIRANLGALQNRLENTISNLQIQSENLQAAESRISDVDVAIEMTEFVRQQIKSSAAVSMLAQANSLPRMAMQLLGG-
         *:*** **.::***  :::**:* :***.*:**:******: ***:: : ** :.:*.:*********::*:.*:*
```

Figure S1. Putative flagellins (FliC) in *D. ferrophilus*.

Organism	fliC gene	Promoter
D. ferrophilus	***Dfe_0352***	***FliA***
	**Dfe_0942**	**unknown**
	**Dfe_0943**	**unknown**
	**Dfe_1910**	**FliA**
	**Dfe_2105**	**FliA**
	**Dfe_2171**	**RpoN**
	**Dfe_2355**	**unknown**
D. desulfuricans	***Dde_1501***	***unknown***
	**Dde_1502**	**unknown**
	**Dde_1570**	**FliA**
	**Dde_1709**	**RpoN**
D. vulgaris	***Dvu_1441***	***RpoN***
	**Dvu_2082**	**FliA**
	**Dvu_2444**	**unknown**

Figure S2. *fliC* genes in *Desulfovibrio* species.

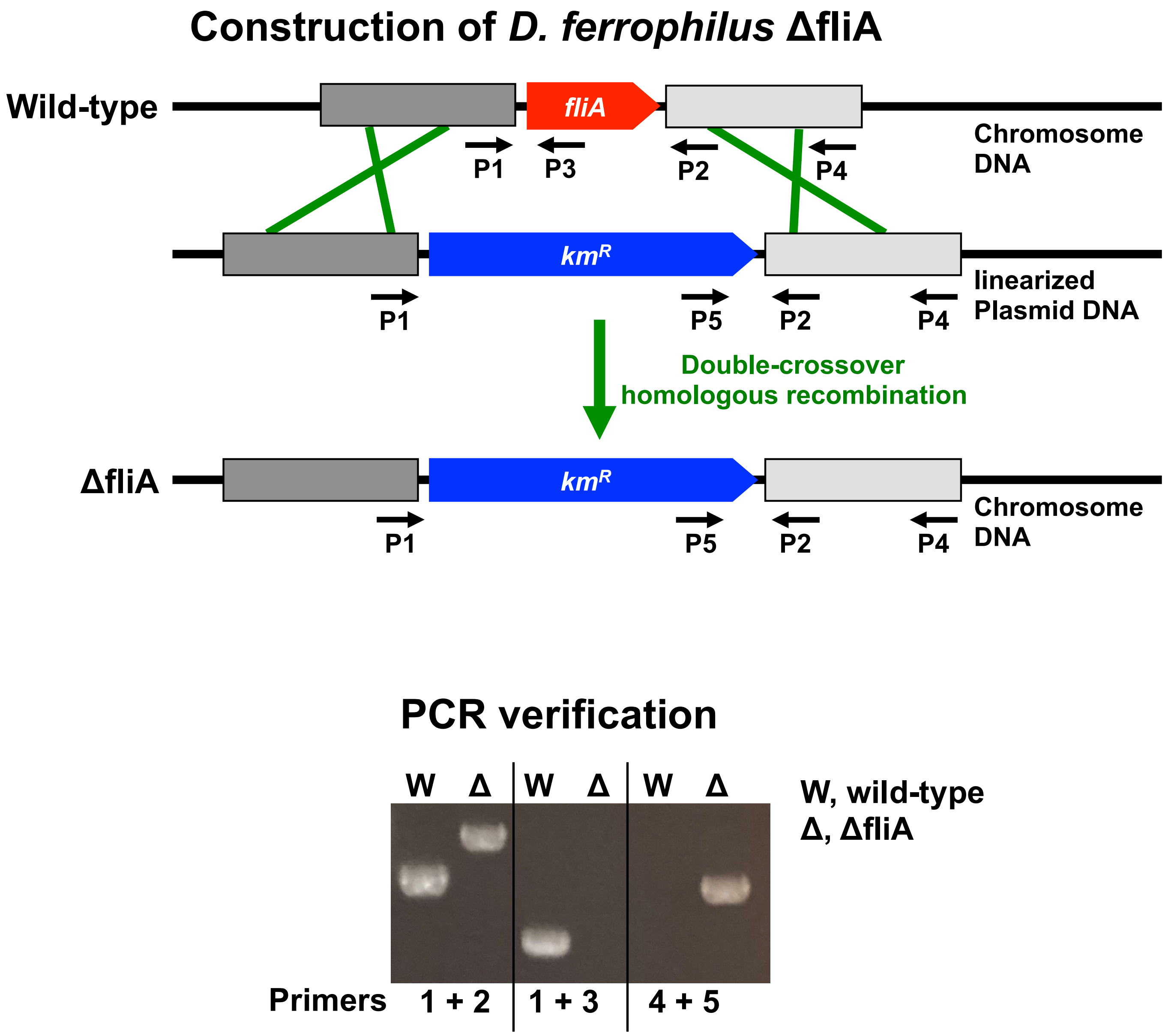

Figure S3. Construction of *D. ferrophilus* ΔfliA strain.

a

```
Gsu_consensus   GCTAAAGTTTT           GCCGATA G
Dvu_concensus     TAAAG               GCCGATA
Dfe_0352_fliC   ACTAAAGTTACCCTGATTC-AAGCCGATAAGACTTGC
Dfe_1910_fliC   CCTAAAGTAACTCCTCCACGAAGACGATGAGACTCCC
Dfe_2105_fliC   CCTAAACTTTTCCCTCCCCGACGCCGAAGAGAAATTA
Dfe_0007_ycgR   CTTAAGATTCTTGCACAGCC-AGCCGATTACGGTATA
Dfe_0889_cheW   GGTAAACTCCTGAGGGGAGCTGCCGATATGACCTGT
Dfe_0231_mcp    GATAAAATGCGTTGCATGG--TACTGATATGTCAGTT
Dfe_1668_mcp    CTTTATATAGCCAAGCATATTTTCCGATAAATCTAAT
Dfe_1893_mcp    GTTAAAGAAGATTTTTGAT-GAGTCGATAAGACTTTG
Dfe_0031_hp     TCTAAAGTGTCGGCTCTGCT-GTCCGATAAGACAGAA
Dfe_2338_hp     CCTAAAGGAACGGCCATAAT-GCCCGATAAGACTTGC
Dfe_consensus     TAAANT              GCCGATAAGACTT
```

b

```
Dfe_0352   ACTAAAGTTACCCTGATTC-AAGCCGATAAGACTTGC
Dfe_1910   CCTAAAGTAACTCCTCCACGAAGACGATGAGACTCCC
Dfe_2105   CCTAAACTTTTCCCTCCCCGACGCCGAAGAGAAATTA
Dde_1570   CCTAAAGTAACGCCG-CACGCCGCCGATAGGAAAAGC
Dvu_2082   CCTAAAGTCCCTACACCA-GCCGCCGATACGACAAGC
```

c

```
Dfe_2171   TTTGGCACTGTGATTGCTTGA
Dde_1709   TTTGGCACCACCTTTGCTCAA
Dvu_1441   ATTGGCATCGTGTTTGCTGAT
             -24         -12
```

Figure S4. Promoter region of genes for flagellar biosynthesis and motility.
(a) Putative FliA-dependent promoter elements in genes for flagellar biosynthesis and motility in *D. ferrophilus*. fliC, flagellin; ycgR, flagellar protein YcgR; cheW, chemotaxis protein W; mcp, methyl-accepting chemotaxis protein; and hp, hypothetical protein.
(b) Putative FliA-dependent promoter elements in *fliC* genes in *Desulfovibrio* species.
(c) Putative RpoN-dependent promoter elements in *fliC* genes in *Desulfovibrio* species. -24/-12 elements are indicated in red.
Gsu, *G. sulfurreducens*; Dfe, *D. ferrophilus*; Dde, *D. desulfuricans*; Dvu, *D. vulgaris*.
Highly conserved sequences are highlighted in yellow.

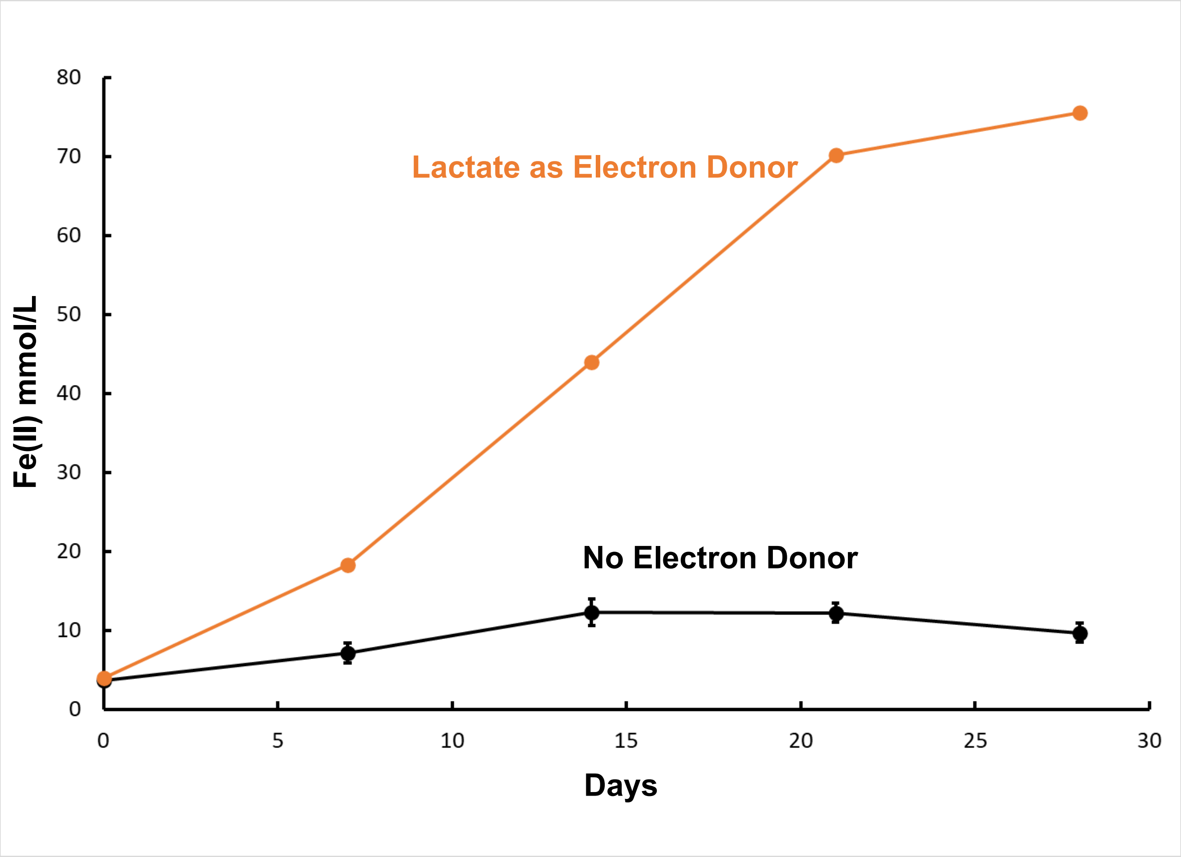

Figure S5. Lack of Fe(III) reduction in the absence of lactate. No electron donor data represents the mean and standard deviation of three replicate cultures.

Table S1. Primers used in this study.
Restriction enzyme sites are underlined. Restriction enzymes used to linearize plasmids for electroporation were indicated in bold.

Name	Sequence	Restriction enzyme
Kanamycin-resistance gene		
km-F	TCTGAATTCCTGACGGAACAGCGGGAAGTC	EcoRI
km-R	TCTAAGCTTTCATAGAAGGCGGCGGTGGAATC	HindIII
km-V	CTTGACGAGTTCTTCTGAGC	
Deletion of DFE_1249 (ΔfliA)		
fliA-up1	ATCTCTAGACGAAGCTCGATGAAGCC	**XbaI**
fliA-up2	TCTGAATTCCAGGGCTCAGTCTTGG	EcoRI
fliA-do1	TCTAAGCTTCACAACTCCACTCTCAAGC	HindIII
fliA-do2	TCTGGTACCAGGAATCGGATCTTGTC	KpnI
fliA-P1	TGCGAACGGCCGTAATCAAC	
fliA-P2	TACCGTCATCGGCTTCCACG	
fliA-P3	GTGACCTCCATGATTTCGTC	
Deletion of DFE_1797-1801 (Δpil5)		
pil5-up1	TCTCTCGAGCTGATGCTGTTCACCAAG	**XhoI**
pil5-up2	TCTAAGCTTGTTGGGACCGTCGGAAC	HindIII
pil5-do1	TCTGAATTCGCACCCAAGTTGCAATAACC	EcoRI
pil5-do2	ATCTCTAGATGGCAACAACGTTGTCGC	XbaI
pil5-P1	TTCCGTAGGTAGAATGTCCG	
pil5-P2	CTCTTGTCATCGGTTACTGC	
pil5-P3	CCGTATTCGATGCTGCACTG	
Deletion of DFE_1987-1988 (Δspp)		
spp-up1	TCTGGTACCTAGATCTGTATCGGTAGC	KpnI
spp-up2	TCTAAGCTTGGAATGAAGTGGTTGCATC	HindIII
spp-do1	TCTGAATTCCAACGGCAATACCCAG	EcoRI
spp-do2	ATCTCTAGACAGGAGGCAATCTCTGC	**XbaI**
spp-P1	AGCCATCTGATTCACTGCAC	
spp-P2	GTGTCGCTGCTGCATCAATC	
spp-P3	CTTGGACTTGATCAGCGTGC	
Deletion of DFE_1992 (Δlpp)		
lpp-up1	TCTGGTACCAAGCGCTAAACACTTAGAC	KpnI
lpp-up2	TCTAAGCTTACTACGTTCCGCTGATGC	HindIII
lpp-do1	TCTGAATTCCTGCCCTAACACGG	EcoRI
lpp-do2	ATCTCTAGATAGTACTCGGGATTGTCG	**XbaI**
lpp-P1	ACCGTTGATGTACAATCCAG	
lpp-P2	TGAACTTGGGCATTGCTGTC	
lpp-P3	GTGTGTCATAGGCTTGCACG	
Deletion of DFE_0448-0450 (Δ448-450)		

448950-up1	TCTGGTACCAGCCCAAACTACTGCGTG	KpnI
448950-up2	TCTAAGCTTCGGCCTCGCAGGCTGCAC	HindIII
448950-do1	TCTGAATTCGGCTGCCATAACAAGCAAC	EcoRI
448950-do2	ATCTCTAGACGTTTACCGTGTGCCGC	**XbaI**
448950-P1	AGATCTATCGCCTGATGGAC	
448950-P2	ATCAGTCTTATGGAGCGCAG	
448950-P3	ATTGACGGCAACCAATGCAG	
Deletion of DFE_0461-0462 (Δ461-462)		
4612-up1	TCTGGTACCGGTTGTTGTCGTCATG	KpnI
4612-up2	TCTAAGCTTGTCAGCAGGTGTATGCC	HindIII
4612-do1	TCTGAATTCTGCAATGCCATAAGGGATAC	EcoRI
4612-do2	ATCTCTAGACTGTTGTGCATGGTGTGAC	**XbaI**
4612-P1	AAGTCCGCAGGCCAGGATAC	
4612-P2	AAGTGTCCGCCACATACAAC	
4612-P3	TTATCCGAGGAATGAGCATC	
Deletion of DFE_0464-0465 (Δ464-465)		
4645-up1	TCTGGTACCTATGCACTGCGGTTCGAC	KpnI
4645-up2	TCTAAGCTTGCCAGCCAGGCCAATACC	HindIII
4645-do1	TCTGAATTCAAGTTTGTTGCCACGGC	EcoRI
4645-do2	ATCTCTAGAGGTATAACAGCTGTAGTCGC	**XbaI**
4645-P1	GCAGATATTGATGTTCGGAC	
4645-P2	TCCAGTACATACGGGCATTC	
4645-P3	GTCCGTAATCAGCAGATTGG	
